# Nomogram prediction model for the risk of intracranial hemorrhagic transformation after intravenous thrombolysis in patients with acute ischemic stroke

**DOI:** 10.3389/fneur.2024.1361035

**Published:** 2024-03-07

**Authors:** Yong Ma, Dong-Yan Xu, Qian Liu, He-Cheng Chen, Er-Qing Chai

**Affiliations:** ^1^Ningxia Medical University, Yinchuan, China; ^2^Cerebrovascular Disease Centre, Gansu Provincial People’s Hospital, Lanzhou, China; ^3^Department of Rehabilitation Medicine, Huashan Hospital, Fudan University, Shanghai, China

**Keywords:** acute ischemic stroke, intravenous thrombolysis, hemorrhagic transformation, nomogram, NLR

## Abstract

**Background:**

Hemorrhagic transformation (HT) after intravenous thrombolysis (IVT) might worsen the clinical outcomes, and a reliable predictive system is needed to identify the risk of hemorrhagic transformation after IVT.

**Methods:**

Retrospective collection of patients with acute cerebral infarction treated with intravenous thrombolysis in our hospital from 2018 to 2022. 197 patients were included in the research study. Multivariate logistic regression analysis was used to screen the factors in the predictive nomogram. The performance of nomogram was assessed on the area under the receiver operating characteristic curve (AUC-ROC), calibration plots and decision curve analysis (DCA).

**Results:**

A total of 197 patients were recruited, of whom 24 (12.1%) developed HT. In multivariate logistic regression model National Institute of Health Stroke Scale (NIHSS) (OR, 1.362; 95% CI, 1.161–1.652; *p* = 0.001), N-terminal pro-brain natriuretic peptide (NT-pro BNP) (OR, 1.012; 95% CI, 1.004–1.020; *p* = 0.003), neutrophil to lymphocyte ratio (NLR) (OR, 3.430; 95% CI, 2.082–6.262; *p* < 0.001), systolic blood pressure (SBP) (OR, 1.039; 95% CI, 1.009–1.075; *p* = 0.016) were the independent predictors of HT which were used to generate nomogram. The nomogram showed good discrimination due to AUC-ROC values. Calibration plot showed good calibration. DCA showed that nomogram is clinically useful.

**Conclusion:**

Nomogram consisting of NIHSS, NT-pro BNP, NLR, SBP scores predict the risk of HT in AIS patients treated with IVT.

## Introduction

Worldwide, neurological disorders are the leading cause of disability and the second leading cause of death, with stroke being the largest cause (1). With the rapid development of interventional techniques and materials in recent years, endovascular intervention has become a primary treatment for acute ischemic stroke, but even so, intravenous thrombolysis (IVT) is now the important and effective treatment for patients within the 4.5-h time window (2, 3). Within 24 h of IVT, a subset of patients may experience a worsening of neurological deficits, which has been described as early neurological deterioration (4), which has been reported to be associated with poor outcomes (5). One of the higher risks of intravenous thrombolysis is hemorrhagic conversion (6). As the risk of hemorrhagic conversion increases, the clinical lethality and disability rates also increase (7). Therefore, it is necessary to develop a predictive model to determine the risk of hemorrhagic conversion after intravenous thrombolysis in patients with AIS.

Currently, nomograms are used as a predictive tool to personalize, visualize and accurately determine such risk. Based on this, the present study aimed to create a nomogram to predict the probability of HT after IVT in Chinese stroke patients.

## Methods

### Study design and data sources

In this study, we consecutively recruited patients diagnosed with AIS from October 2018 to October 2022 in Gansu Provincial People’s Hospital. Included patients met the following criteria: (1) age ≥ 18 years; (2) diagnosis of acute ischemic stroke; (3) time to treatment initiation <4.5 h; and (4) patients receiving intravenous thrombolysis with rt-PA. Patients who met the following criteria were excluded: (1) intra-arterial thrombolysis or endovascular thrombolysis after intravenous thrombolysis; (2) those diagnosed with intracranial hemorrhage, including subarachnoid hemorrhage, parenchymal hemorrhage, intraventricular hemorrhage, epidural hemorrhage and so on; (3) incomplete clinical data. The study was approved by the Ethics Committee of Gansu Provincial People’s Hospital (Approval No. 2023-350), Written informed consent was waived due to the retrospective nature of this study. All procedures performed in the study complied with the 1964 Declaration of Helsinki and its subsequent amendments or similar ethical standards.

### Baseline data collection

Demographic characteristics, medical history, and clinical and laboratory data were obtained at admission. Stroke severity was assessed by National Institutes of Health Stroke Scale (NIHSS) score. Laboratory data included baseline blood glucose, systolic blood pressure (SBP), diastolic blood pressure (DBP), neutrophil-to-lymphocyte ratio (NLR), high-density lipoproteins (HDL), low-density lipoproteins (LDL), triglycerides (TG), N-terminal pro-brain natriuretic peptide (NT-pro BNP), and total cholesterol (TC), et al. The NLR values were calculated as neutrophil count/lymphocyte count.

### Definition of hemorrhagic transformation

Hemorrhagic transformation (HT) was defined as any type of intracranial hemorrhage detected by follow-up CT or MRI within 22–36 h after intravenous thrombolysis, according to the criteria of the European Cooperative Acute Stroke Study II (8). All images were judged by two experienced neurologists without knowledge of the clinical data and final diagnosis.

### Statistical analysis

Statistical analyses Descriptive analyses were as follows: Continuous variables were expressed as having mean ± standard deviation or median (interquartile range); Categorical variables are described as numbers with percentages. Differences between groups with and without HT were investigated using Mann–Whitney U tests or t tests for appropriate continuous variables. Where appropriate, differences between the two groups of categorical variables were analyzed by Fisher’s exact test or *χ*^2^ test.

To construct nomograms, we used multivariate logistic regression analyses to identify independent factors for HT, and all variables with *p* values <0.05 in univariate analyses were included. Variables with *p* values <0.05 in multivariate logistic regression were entered to generate predictive models. Regression coefficients and 95% confidence intervals (CI) for each variable in the model were calculated for the odds ratio (OR). The regression coefficients for each variable in the model were used to calculate the corresponding scores in the scale and ultimately to obtain the scoring system. The discriminative power of the Nomogram was assessed by calculating the area under the receiver operating characteristic curve (AUC-ROC). The calibration of the prediction model describing the agreement between observed and predicted probabilities based on nomograms was tested using 1,000 resampled calibration plots. All statistical analyses were performed using statistical methods Software SPSS version 26.0 (IBM, New York, NY) and R version 4.3 (R Foundation, Vienna, Austria).

## Results

Patients a total of 243 patients with ischemic stroke were treated with IVT. Patients who underwent intra-arterial thrombolysis (*n* = 7) or endovascular thrombectomy (*n* = 29) and those who lacked complete data (*n* = 10) were excluded. As shown in Table 1, 24 (11%) of the baseline profile characteristics were post-thrombolytic HT. Table 2 univariate logistic analysis showing NIHSS score, NLR, SBP, and NT-pro BNP (*p* < 0.05). After multivariate logistic analysis, NIHSS score, NLR, SBP, and NT-pro BNP were shown to be independent predictors of HT after intravenous thrombolysis in patients with ischemic stroke.

**Table 1:** Baseline characteristics of AIS patients with IVT.

Variable	With HT (*n* = 24)	Without HT (*n* = 173)	Overall (*n* = 197)	*p*-value
Demographics				
Age, years	69.83 ± 11.82	67.86 ± 12.28	68.10 ± 12.21	0.459
Male, n (%)	17 (70.8)	117 (67.6)	134 (68.0)	0.753
Medical history, n (%)				
Hypertension	16 (66.7)	112 (64.7)	128 (65.0)	0.853
Diabetes mellitus	6 (25.0)	36 (20.8)	42 (21.3)	0.639
Coronary heart disease	5 (20.8)	30 (17.3)	35 (17.8)	0.675
Smoking	7 (29.2)	43 (24.9)	50 (25.4)	0.649
Drinking	6 (25.0)	30 (17.3)	36 (18.3)	0.363
Previous stroke	5 (20.8)	29 (16.8)	34 (17.3)	0.621
Clinical data				
SBP, mmHg	164.88 ± 22.94	151.35 ± 19.51	152.99 ± 20.38	0.002
DBP, mmHg	89.50 ± 22.49	87.72 ± 17.44	87.93 ± 18.08	0.652
NIHSS, score	10.50 [7.00, 15.25]	6.00 [5.00, 8.00]	7.00 [5.00, 9.00]	<0.001
Time from onset to treatment, min	144.50 [112.50, 215.25]	171.00 [136.00, 211.00]	170.00 [125.00, 212.00]	0.651
Laboratory data				
Glucose, mmol/L	7.70 ± 2.24	7.17 ± 2.27	7.23 ± 2.26	0.286
NEUT, %	69.83 ± 11.55	67.90 ± 13.99	68.13 ± 13.70	0.519
NLR	4.86 [3.40, 6.03]	3.07 [2.39, 3.64]	3.21 [2.45, 3.92]	<0.001
Platelet, 10∧9/L	172.50 [142.75, 211.25]	192.00 [160.00, 230.00]	191.00 [159.00, 229.00]	0.168
PT, s	13.78 [12.63, 15.12]	12.90 [10.89, 15.19]	13.12 [11.14, 15.19]	0.118
APTT, s	27.91 [24.41, 31.13]	29.04 [25.58, 31.81]	28.84 [25.50, 31.81]	0.384
INR	0.98 [0.90, 1.03]	0.98 [0.92, 1.04]	0.98 [0.92, 1.04]	0.574
TG, mmol/L	1.91 ± 0.70	1.79 ± 0.72	1.81 ± 0.71	0.443
TC, mmol/L	3.38 [2.01, 4.94]	3.60 [2.52, 4.50]	3.60 [2.34, 4.51]	0.756
HDL, mmol/L	1.40 [0.97, 2.19]	1.77 [1.24, 2.24]	1.72 [1.20, 2.24]	0.383
LDL, mmol/L	3.09 [2.57, 3.79]	3.10 [2.42, 3.90]	3.10 [2.43, 3.89]	0.598
NT-pro BNP	302.67 ± 127.53	205.39 ± 100.24	217.25 ± 108.37	<0.001

AIS, acute ischemic stroke; IVT, intravenous thrombolysis; HT, Hemorrhagic transformation; SBP, systolic blood pressure; DBP, diastolic blood pressure; NIHSS, National Institutes of Health Stroke Scale; NEUT, Neutrophilic granulocyte percentage; NLR, neutrophil-to-lymphocyte ratio; PT, prothrombin time; APTT, activated partial thromboplastin time; INR, International normalized ratio; TG, triglyceride; TC, total cholesterol; HDL, High-density lipoprotein; LDL, low-density lipoprotein; NT-pro BNP, the N-terminal of the prohormone brain natriuretic peptide.

**Table 2:** Univariable and multivariable analyses of HT in AIS patients with intravenous thrombolysis.

Variable	Crude OR (95%CI)	Uni -*p* value	Adj OR (95%CI)	multi-*p* value
Age	1.014 [0.979, 1.052]	0.457		
Male	1.162 [0.472, 3.152]	0.753		
Hypertension	1.089 [0.452, 2.820]	0.853		
Diabetes mellitus	1.269 [0.434, 3.276]	0.639		
Coronary heart disease	1.254 [0.392, 3.409]	0.675		
Smoking	1.245 [0.456, 3.099]	0.65		
Drinking	1.589 [0.540, 4.153]	0.366		
Previous stroke	1.307 [0.408, 3.560]	0.622		
SBP	1.035 [1.012, 1.060]	0.003	1.039 [1.009, 1.075]	0.016
DBP	1.006 [0.982, 1.030]	0.65		
NIHSS	1.335 [1.194, 1.519]	<0.001	1.362 [1.161, 1.652]	0.001
Time from onset to treatment	0.998 [0.991, 1.006]	0.674		
GLU	1.099 [0.915, 1.301]	0.287		
NEUT	1.011 [0.979, 1.044]	0.517		
NLR	2.712 [1.889, 4.107]	<0.001	3.430 [2.082, 6.262]	<0.001
PLT	0.994 [0.986, 1.002]	0.146		
PT	1.125 [0.980, 1.303]	0.104		
APTT	0.961 [0.876, 1.050]	0.381		
INR	0.204 [0.001, 29.445]	0.531		
TG	1.266 [0.694, 2.318]	0.441		
TC	0.949 [0.701, 1.279]	0.733		
HDL	0.760 [0.405, 1.390]	0.379		
LDL	1.143 [0.749, 1.763]	0.537		
NT-pro BNP	1.008 [1.004, 1.013]	<0.001	1.012 [1.004, 1.020]	0.003

AIS, acute ischemic stroke; IVT, intravenous thrombolysis; HT, Hemorrhagic transformation; CI, confidence interval; OR, odds ratio; SBP, systolic blood pressure; DBP, diastolic blood pressure; NIHSS, National Institutes of Health Stroke Scale; NEUT, Neutrophilic granulocyte percentage; NLR, neutrophil-to-lymphocyte ratio; PT, prothrombin time; APTT, activated partial thromboplastin time; INR, International normalized ratio; TG, triglyceride; TC, total cholesterol; HDL, High-density lipoprotein; LDL, low-density lipoprotein; NT-pro BNP, the N-terminal of the prohormone brain natriuretic peptide.

A nomogram of the HT predictive model was created based on these risk factors. The score for each independent predictor is the score corresponding to the upper scale, and the total score for each subject is the sum of the scores for each independent predictor. The total number of points corresponding to the HT risk axis is the risk of HT. The higher the total score, the higher the risk of HT. Internal validation of the Nomogram was performed by repeated sampling 1,000 times using the Bootstrap method.

**Figure 1:**
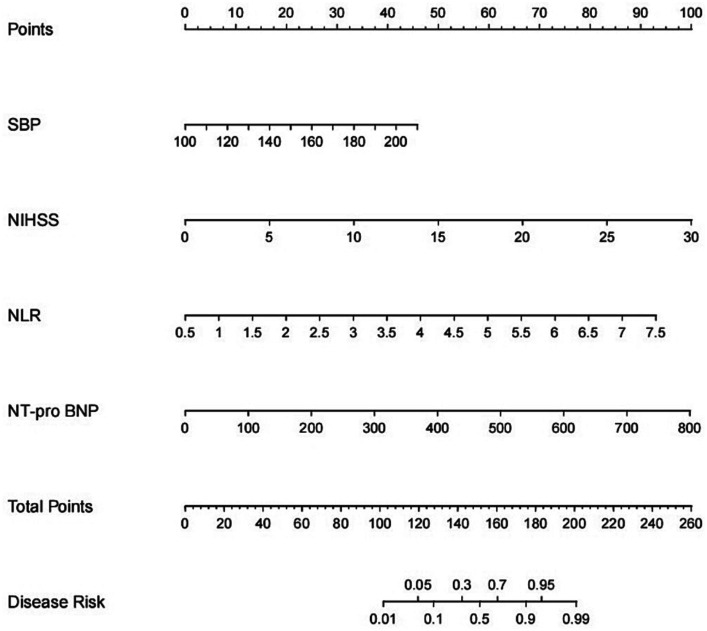
Nomogram for predicting HT after IVT. A line graph consisting of the SBP, NIHSS, NLR, NT-pro BNP. A vertical line was drawn from the axis corresponding to each predictor until the top line labeled “points” was reached totaling the number of points for all predictors, and then a line was drawn down the axis labeled “total points” until it intersected the risk of intracranial hemorrhagic transformation after intravenous thrombolysis in patients with acute ischemic stroke.

**Figure 2:**
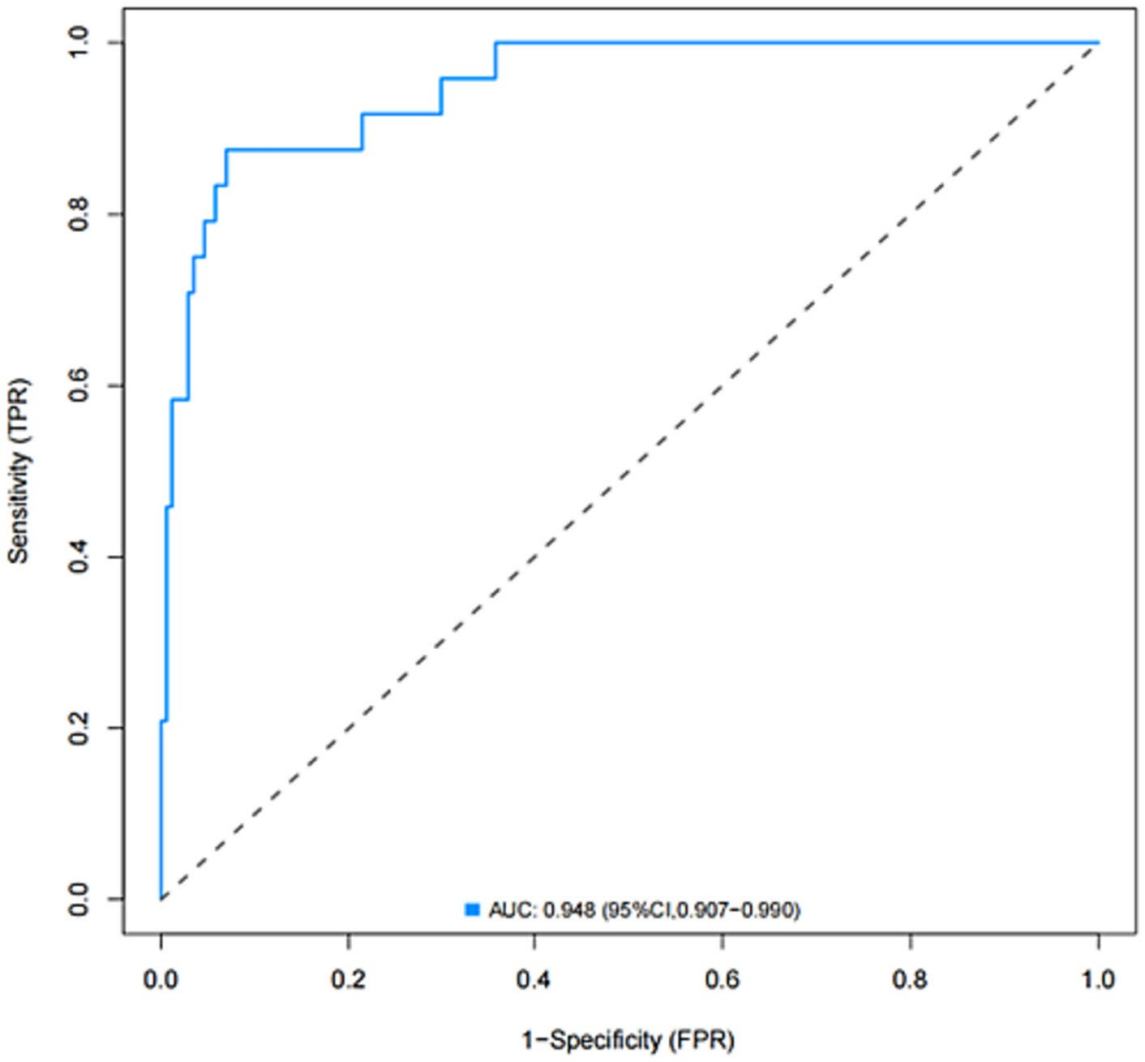
Receiver operating characteristic (ROC) curve of the nomogram for predicting the risk of HT after IVT.

**Figure 3:**
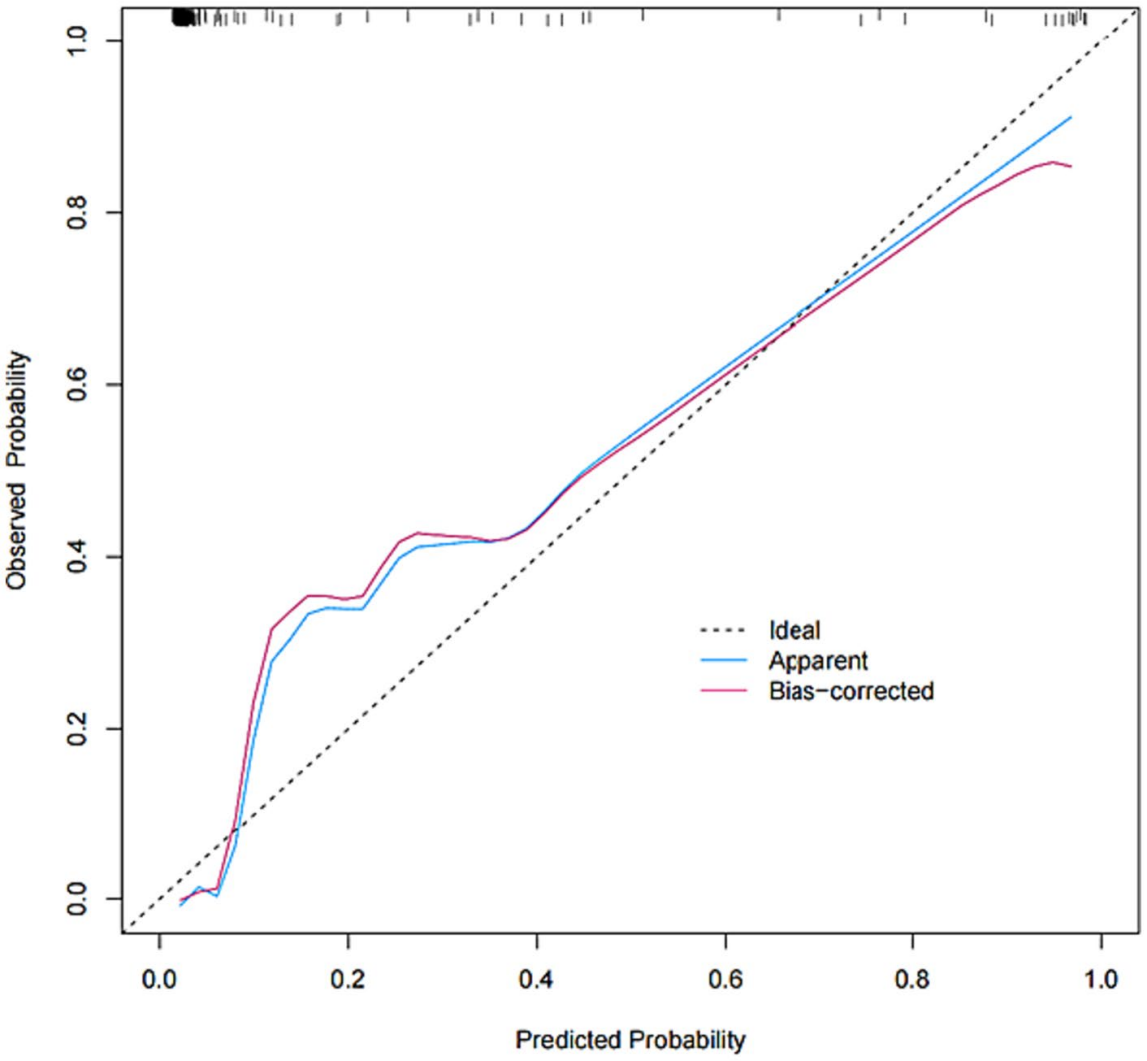
Calibration plot for predicting HT after IVT.

**Figure 4:**
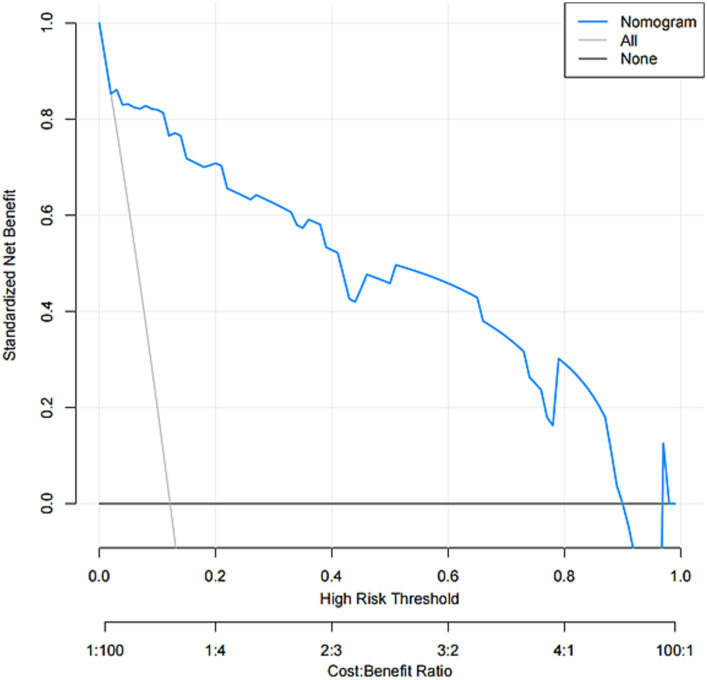
Decision curve analysis for the nomogram.

The model was created by combining the independent predictor values as described above and shown as a nomogram in Figure 1. The score for each predictor in the Nomo plot is determined by drawing a vertical line between the predictor fold and the preliminary score line. The total score is calculated by totaling the scores for each predictor, and the corresponding HT prediction probabilities are obtained by drawing a vertical line between the total score and the probability line. The AUC-ROC for the prediction model was (Figure 2). In addition, the calibration curves of the nomograms for the likelihood of HT in patients showed good agreement (Figure 3), predicting the model HT probability. The calibration curves are shown in Figure 3. The calibration curves used to estimate HT showed no significant deviation from perfect match and good agreement between predicted and actual results. The analysis of the decision curve (DCA) (Figure 4) showed that clinical decision making based on the predictive model was beneficial and implied the practical clinical application and operability of the predictive model.

## Discussion

In this study, we found that NIHSS score, NLR, SBP, and NT-pro BNP were independent predictors of HT. Based on these four independent factors, we constructed a predictive nomogram. This model can help us to predict the probability of HT in acute ischemic stroke patients treated with IVT. The developed nomogram showed good discrimination and calibration. In addition, DCA results showed that the developed nomogram had a significant net benefit in predicting the risk of cerebral hemorrhage.

Firstly, similar to previous studies, the higher the pre-treatment NIHSS score, the greater the risk of HT (9, 10). One article showed a 1.6-fold increased risk of HT in patients with acute cerebral infarction with an NIHSS score of 7–12 and a 2.22-fold increased risk of HT in patients with a baseline NIHSS score of ≥13 (11). In this study, it was shown that patients with high NIHSS scores also had a higher risk of HT. This is because the higher the NIHSS score, the larger the area of cerebral infarction and oedema in the patient, the higher the risk of HT (12, 13). The above previous studies have shown that high NIHSS score is closely associated with hemorrhagic transformation, which is consistent with the findings of the current study.

NLR is easily accessible in the clinic and reproduces new biomarkers of inflammation (14). It plays a crucial role in HT due to the inflammatory response of migrating inflammatory cells leading to disruption of blood–brain barrier integrity (15). Neutrophils can increase the permeability of the blood–brain barrier by releasing, among other things, associated cytokines, whereas activation of lymphocytes can reduce blood–brain barrier disruption. Due to the balance between neutrophils and lymphocytes, NLR is considered a biomarker of systemic inflammation. High NLR (≥4.255) on admission has been reported to increase the risk of HT in patients with AIS after IVT (16). A clinical study by Guo et al. reported that the dynamics of HT were associated with IVT in patients with acute ischemic stroke (17). The underlying mechanism by which NLR increases the risk of cerebral hemorrhage in patients with AIS treated with IVT has not been elucidated. A plausible explanation may be that NLR influences outcome as it is associated with inflammatory destruction of neutrophils and reduced lymphocyte protection (18).

Hypertension was found to be a risk factor for hemorrhagic transformation after intravenous thrombolysis. Similar results were obtained by He et al. who suggested that increased SBP mediates brain–blood barrier damage and upregulation of aquaporin Protein-4 via oxidative stress, leading to an increased risk of neurological deterioration (19). Meanwhile, hypertension impairs collateral circulation, reduces the ability of brain tissue to maintain adequate oxygenation during cerebral artery occlusion, and promotes the accumulation of reactive oxygen species and the release of inflammatory factors, leading to further damage to the blood–brain barrier, which in turn leads to hemorrhagic transformation after thrombolysis (20).

There is still some controversy about whether a history of previous hypertension serves as a risk factor for hemorrhagic transformation (21, 22), which is because chronic hypertension leads to increased permeability of the blood–brain barrier, impaired reperfusion of blood flow, and damage to the inner wall of blood vessels leading to blood leakage, secondary to hemorrhagic transformation. And the history of hypertension was not found to affect HT during the study of this paper, which needs to be supported by subsequent studies.

Another major finding of this paper is the correlation between elevated levels of NT-pro BNP and hemorrhagic conversion in stroke patients treated with intravenous thrombolysis. NT-pro BNP is released from ventricular myocardium with stretching (23). Several studies have also shown that the brain secretes NT-pro BNP and that the concentration of NT-pro BNP in the cerebrospinal fluid may be greatly increased after brain injury (24, 25). Hemorrhagic transformation is a major complication in stroke patients treated with intravenous thrombolysis. The association between elevated NT-pro BNP levels and cerebral hemorrhage has been demonstrated It has been demonstrated (26) that elevated NT-pro BNP levels are associated with increased hematoma volume and a poor prognosis (27, 28). Our findings suggest that NT-pro BNP levels are independently associated with hemorrhagic hypertension in stroke patients receiving intravenous thrombolytic therapy for transformation. Another potential reason for elevated NT-pro BNP levels in hemorrhagic transformation may be that hemorrhagic transformation exacerbates ischemic stroke-induced neurological damage (29), and may also exacerbate stroke-induced cardiac dysfunction in the same way (30, 31). However, whether thrombolytic therapy affects NT-pro BNP levels remains unclear. Future studies are needed to further elucidate the mechanism of elevated NT-pro BNP levels in stroke patients receiving intravenous thrombolytic therapy.

## Conclusion

Our study presents a novel and practical nomogram of NIHSS, NT-pro BNP, NLR, SBP that can well predict the probability of HT after intravenous thrombolysis in ischemic stroke patients. The qualitative and discriminative properties of the graph were verified in an internal validation. The graph can be used to predict the probability of HT after IVT and to help clinicians assess whether to continue IVT in patients at high risk for HT. However, further studies are needed to confirm the validity of the nomogram.

### Strengths and limitations

The strengths of our study are as follows: The prognostic factors included in the nomogram can be easily and quickly obtained at the time of admission. Still, there are some limitations to our study. First of all, the sample size of this study is small, and there is a certain degree of selectivity bias. Second, our data came from a single-center retrospective analysis, which may limit the statistical power of the results. Finally, our model has not yet been validated in an external queue.

## Data availability statement

The raw data supporting the conclusions of this article will be made available by the authors, without undue reservation.

## Ethics statement

The studies involving humans were approved by the Ethics Committee of Gansu Provincial People’s Hospital (Approval No. 2023-350). The studies were conducted in accordance with the local legislation and institutional requirements. Written informed consent from the patients/participants or patients/participants’ legal guardian/next of kin was not required to participate in this study in accordance with the national legislation and the institutional requirements.

## Author contributions

YM: Conceptualization, Data curation, Software, Validation, Visualization, Writing – original draft, Writing – review & editing. D-YX: Formal analysis, Software, Writing – original draft. QL: Software, Visualization, Writing – original draft. H-CC: Conceptualization, Methodology, Validation, Writing – original draft, Writing – review & editing. E-QC: Conceptualization, Methodology, Software, Writing – original draft, Writing – review & editing.

## References

[1] GBD 2016 Neurology Collaborators. Global, regional, and national burden of neurological disorders, 1990-2016: a systematic analysis for the global burden of disease study 2016. Lancet Neurol. (2019) 18:459–80. doi: 10.1016/S1474-4422(18)30499-X 30879893 PMC6459001

[2] Powers WJ Rabinstein AA Ackerson T Adeoye OM Bambakidis NC Becker K. et al; American Heart Association stroke council. 2018 guidelines for the early Management of Patients with Acute Ischemic Stroke: A guideline for healthcare professionals from the American Heart Association/American Stroke Association. Stroke. (2018) 49:e46–e110. doi: 10.1161/STR.0000000000000158 29367334

[3] Elfil M Bahbah EI Bayoumi A Aladawi M Eldokmak M Salem MM. Repeated mechanical thrombectomy for recurrent large vessel occlusion: A systematic review and meta-analysis. Interv Neuroradiol. (2022). doi: 10.1177/15910199221134307, [Online ahead of print] PMC11569475 36285483

[4] Seners P Hurford R Tisserand M Turc G Legrand L Naggara O. Is unexplained early neurological deterioration after intravenous thrombolysis associated with Thrombus extension? Stroke. (2017) 48:348–52. doi: 10.1161/STROKEAHA.116.015414, PMID: 28034965

[5] Mori M Naganuma M Okada Y Hasegawa Y Shiokawa Y Nakagawara J. Early neurological deterioration within 24 hours after intravenous rt-PA therapy for stroke patients: the stroke acute management with urgent risk factor assessment and improvement rt-PA registry. Cerebrovasc Dis. (2012) 34:140–6. doi: 10.1159/000339759, PMID: 22854333

[6] Aoki J Iguchi Y Urabe T Yamagami H Todo K Fujimoto S. Acute aspirin plus cilostazol dual therapy for noncardioembolic stroke patients within 48 hours of symptom onset. J Am Heart Assoc. (2019) 8:e012652. doi: 10.1161/JAHA.119.012652, PMID: 31347430 PMC6761671

[7] Zhao Y Yang W Tan Z Wang W Xiao W Zeng J. Clopidogrel loading dose versus maintenance dose to treat patients with acute ischaemic stroke in China (CLASS-China): results from a prospective double-blind randomised clinical trial. Stroke Vasc Neurol. (2017) 2:118–23. doi: 10.1136/svn-2017-000072, PMID: 28989802 PMC5628375

[8] Larrue V von Kummer RR Müller A Bluhmki E. Risk factors for severe hemorrhagic transformation in ischemic stroke patients treated with recombinant tissue plasminogen activator: a secondary analysis of the European-Australasian acute stroke study (ECASS II). Stroke. (2001) 32:438–41. doi: 10.1161/01.str.32.2.438 11157179

[9] Tanaka K Matsumoto S Furuta K Yamada T Nagano S Takase KI. Differences between predictive factors for early neurological deterioration due to hemorrhagic and ischemic insults following intravenous recombinant tissue plasminogen activator. J Thromb Thrombolysis. (2020) 49:545–50. doi: 10.1007/s11239-019-02015-4, PMID: 31848874 PMC7182629

[10] Tong X George MG Yang Q Gillespie C. Predictors of in-hospital death and symptomatic intracranial hemorrhage in patients with acute ischemic stroke treated with thrombolytic therapy: Paul Coverdell acute stroke registry 2008-2012. Int J Stroke. (2014) 9:728–34. doi: 10.1111/ijs.12155 24024962 PMC4451118

[11] Mazya M Egido JA Ford GA Lees KR Mikulik R Toni D. Predicting the risk of symptomatic intracerebral hemorrhage in ischemic stroke treated with intravenous alteplase: safe implementation of treatments in stroke (SITS) symptomatic intracerebral hemorrhage risk score. Stroke. (2012) 43:1524–31. doi: 10.1161/STROKEAHA.111.644815 22442178

[12] Michel S Buchholz S Buech J Veit T Fabry T Abicht J. Bridging patients in cardiogenic shock with a paracorporeal pulsatile biventricular assist device to heart transplantation-a single-Centre experience. Eur J Cardiothorac Surg. (2022) 61:942–9. doi: 10.1093/ejcts/ezab547, PMID: 35020902

[13] Mihatov N Mosarla RC Kirtane AJ Parikh SA Rosenfield K Chen S. Outcomes associated with peripheral artery disease in myocardial infarction with cardiogenic shock. J Am Coll Cardiol. (2022) 79:1223–35. doi: 10.1016/j.jacc.2022.01.037, PMID: 35361344 PMC9172933

[14] Ma G Pan Z Kong L Du G. Neuroinflammation in hemorrhagic transformation after tissue plasminogen activator thrombolysis: potential mechanisms, targets, therapeutic drugs and biomarkers. Int Immunopharmacol. (2021) 90:107216. doi: 10.1016/j.intimp.2020.107216, PMID: 33296780

[15] Jickling GC Liu D Stamova B Ander BP Zhan X Lu A. Hemorrhagic transformation after ischemic stroke in animals and humans. J Cereb Blood Flow Metab. (2014) 34:185–99. doi: 10.1038/jcbfm.2013.203, PMID: 24281743 PMC3915212

[16] Liu YL Lu JK Yin HP Xia PS Qiu DH Liang MQ. High neutrophil-to-lymphocyte ratio predicts hemorrhagic transformation in acute ischemic stroke patients treated with intravenous thrombolysis. Int J Hypertens. (2020) 2020:5980261–6. doi: 10.1155/2020/5980261, PMID: 32181011 PMC7064843

[17] Guo Z Yu S Xiao L Chen X Ye R Zheng P. Dynamic change of neutrophil to lymphocyte ratio and hemorrhagic transformation after thrombolysis in stroke. J Neuroinflammation. (2016) 13:199. doi: 10.1186/s12974-016-0680-x, PMID: 27561990 PMC5000487

[18] Kim JY Park J Chang JY Kim SH Lee JE. Inflammation after ischemic stroke: the role of leukocytes and glial cells. Exp Neurobiol. (2016) 25:241–51. doi: 10.5607/en.2016.25.5.241, PMID: 27790058 PMC5081470

[19] He Y Yang Q Liu H Jiang L Liu Q Lian W. Effect of blood pressure on early neurological deterioration of acute ischemic stroke patients with intravenous rt-PA thrombolysis may be mediated through oxidative stress induced blood-brain barrier disruption and AQP4 upregulation. J Stroke Cerebrovasc Dis. (2020) 29:104997. doi: 10.1016/j.jstrokecerebrovasdis.2020.104997, PMID: 32689627

[20] Wu D Liu Y. FM combined with NIHSS score contributes to early AIS diagnosis and differential diagnosis of cardiogenic and non-cardiogenic AIS. Clin Appl Thromb Hemost. (2021) 27:10760296211000129. doi: 10.1177/10760296211000129, PMID: 33724895 PMC7970226

[21] Al-Kawaz M Cho SM Gottesman RF Suarez JI Rivera-Lara L. Impact of cerebral autoregulation monitoring in cerebrovascular disease: a systematic review. Neurocrit Care. (2022) 36:1053–70. doi: 10.1007/s12028-022-01484-5, PMID: 35378665

[22] Al-Mufti F Amuluru K Changa A Lander M Patel N Wajswol E. Traumatic brain injury and intracranial hemorrhage-induced cerebral vasospasm: a systematic review. Neurosurg Focus. (2017) 43:E14. doi: 10.3171/2017.8.FOCUS17431, PMID: 29088959

[23] Daniels LB Maisel AS. Natriuretic peptides. J Am Coll Cardiol. (2007) 50:2357–68. doi: 10.1016/j.jacc.2007.09.021 18154959

[24] Manea MM Comsa M Minca A Dragos D Popa C. Brain-heart axis—review article. J Med Life. (2015) 8:266–71. PMID: 26351525 PMC4556904

[25] Ru D Yan Y Li B Shen X Tang R Wang E. BNP and NT-pro BNP concentrations in paired cerebrospinal fluid and plasma samples of patients with traumatic brain injury. J Surg Res. (2021) 266:353–60. doi: 10.1016/j.jss.2021.04.018, PMID: 34087618

[26] Di Castelnuovo A Veronesi G Costanzo S Zeller T Schnabel RB de Curtis A. NT-proBNP (N-terminal pro-B-type natriuretic peptide) and the risk of stroke. Stroke. (2019) 50:610–7. doi: 10.1161/STROKEAHA.118.023218 30786848

[27] Li F Chen QX Xiang SG Yuan SZ Xu XZ. The role of N-terminal pro-brain natriuretic peptide in evaluating the prognosis of patients with intracerebral hemorrhage. J Neurol. (2017) 264:2081–7. doi: 10.1007/s00415-017-8602-0, PMID: 28840579

[28] Li F Chen QX Xiang SG Yuan SZ Xu XZ. N-terminal pro-brain natriuretic peptide concentrations after hypertensive intracerebral hemorrhage: relationship with hematoma size, hyponatremia, and intracranial pressure. J Intensive Care Med. (2018) 33:663–70. doi: 10.1177/0885066616683677, PMID: 28040989

[29] Hajdinjak E Klemen P Grmec S. Prognostic value of a single prehospital measurement of N-terminal pro-brain natriuretic peptide and troponin T after acute ischaemic stroke. J Int Med Res. (2012) 40:768–76. doi: 10.1177/147323001204000243, PMID: 22613442

[30] Chen Z Venkat P Seyfried D Chopp M Yan T Chen J. Brain-heart interaction: cardiac complications after stroke. Circ Res. (2017) 121:451–68. doi: 10.1161/CIRCRESAHA.117.311170, PMID: 28775014 PMC5553569

[31] Battaglini D Robba C Lopes da Silva A Dos Santos Samary C Leme Silva P Dal Pizzol F. Brain-heart interaction after acute ischemic stroke. Crit Care. (2020) 24:163. doi: 10.1186/s13054-020-02885-8 32317013 PMC7175494

